# Hi-GeoMVP: a hierarchical geometry-enhanced deep learning model for drug response prediction

**DOI:** 10.1093/bioinformatics/btae204

**Published:** 2024-04-13

**Authors:** Yurui Chen, Louxin Zhang

**Affiliations:** Department of Mathematics and the Centre for Data Science and Machine Learning, National University of Singapore, Singapore 119076, Singapore; Department of Mathematics and the Centre for Data Science and Machine Learning, National University of Singapore, Singapore 119076, Singapore

## Abstract

**Motivation:**

Personalized cancer treatments require accurate drug response predictions. Existing deep learning methods show promise but higher accuracy is needed to serve the purpose of precision medicine. The prediction accuracy can be improved with not only topology but geometrical information of drugs.

**Results:**

A novel deep learning methodology for drug response prediction is presented, named Hi-GeoMVP. It synthesizes hierarchical drug representation with multi-omics data, leveraging graph neural networks and variational autoencoders for detailed drug and cell line representations. Multi-task learning is employed to make better prediction, while both 2D and 3D molecular representations capture comprehensive drug information. Testing on the GDSC dataset confirms Hi-GeoMVP’s enhanced performance, surpassing prior state-of-the-art methods by improving the Pearson correlation coefficient from 0.934 to 0.941 and decreasing the root mean square error from 0.969 to 0.931. In the case of blind test, Hi-GeoMVP demonstrated robustness, outperforming the best previous models with a superior Pearson correlation coefficient in the drug-blind test. These results underscore Hi-GeoMVP’s capabilities in drug response prediction, implying its potential for precision medicine.

**Availability and implementation:**

The source code is available at https://github.com/matcyr/Hi-GeoMVP

## 1 Introduction

Predicting individual patient responses to specific drugs is a critical aspect of cancer treatment. However, direct prediction of drug response in patients often proves impractical due to ethical considerations, invasiveness, and prohibitive costs. Hence, preclinical models like tumor samples and cancer cell lines are used as alternatives. Cancer cell lines, comprehensively profiled at the transcriptomic, epigenomic, and proteomic levels, offer valuable insights into drug response (Weinstein 2012). As a result, predicting drug response in cell lines has emerged as a vital stage in personalized cancer treatment (Gillet *et al.* 2013). The advent of high-throughput phenotypic drug screening has enabled efficient and systematic testing of a multitude of compounds on cancer cell lines. Two pivotal datasets, the Genomics of Drug Sensitivity in Cancer (GDSC) (Iorio *et al.* 2016) and the Cancer Cell Line Encyclopedia (Barretina *et al.* 2012), have significantly impacted the evolution of machine learning techniques for drug response prediction.

Advancements in data collection have facilitated the profiling of cancer cell lines at the molecular level, enabling researchers to construct efficient computational models for accurate drug response prediction. Various predictive methodologies have been proposed, including matrix-factorization (MF)-based methods (Wang *et al.* 2017), regression methods (Iorio *et al.* 2016), bayesian inference methods (Wang *et al.* 2020), and deep learning (DL) methods (Liu *et al.* 2020, Nguyen *et al.* 2022). These methods have been systematically evaluated and compared in several survey papers (Chen and Zhang 2021, 2022, Shen *et al.* 2023). MF-based models have been particularly effective. To improve performance, researchers have considered different relationships between drugs and cell lines. For example, the similarity-regularized matrix factorization (SRMF) postulates that drugs or cell lines with similar patterns will likely exhibit similar response values. MF methods can handle sparse data effectively, even when missing pairs exist in the drug response matrix. However, as inductive methods, MF methods can only generate latent representation on seen cell lines and drugs in the training set, thus it is not efficient to make prediction on the new drug, new cell lines, or both.

DL methods have shown potential in predicting drug responses due to their capability to handle high-dimensional features and model non-linear relationships between drugs and cell lines (Chen and Zhang 2022). Models that integrate multiple types of omics data, such as genomic mutations and gene expression, have demonstrated superior prediction accuracy compared to when these models are constrained to using only a single type of omics data (Liu *et al.* 2020, Zhu *et al.* 2022).

A drug is often characterized through a molecular graph treating each atom as a node and each chemical bond as an edge, and the graph neural network (GNN) is applied. GraphDRP (Nguyen *et al.* 2022) tests the efficacy of three distinct GNN models: GCN (Gilmer *et al.* 2017), graph attention networks (GATs) (Veličković *et al.* 2018), and graph isomorphism network (GIN) (Hu *et al.* 2020).

Although these models provide innovative solutions, they do possess limitations. Firstly, a 2D molecular graph, using both the atom attributes and the connections between atom pairs, represents only the topology of the molecule without capturing intricate 3D geometric information or the spatial arrangements of atoms within the drugs. For example, cis-Platin, an FDA-approved drug, is used to treat various cancers. But, trans-Platin has no anticancer activity despite sharing the same topology (Dasari and Tchounwou 2014).

Moreover, some cancer drugs target specific biomarkers based on distinct omics data, such as Trastuzumab’s reliance on gene expression data for its action on the HER2 gene and Vemurafenib’s targeting of the V600E mutation in the BRAF gene (Chapman *et al.* 2011). These interactions are not explicitly modeled, potentially reducing the accuracy of the model’s predictions.

To address the limitations of previous studies and improve the prediction accuracy, we propose the Hierarchical Geometry-enhanced Multi-View drug response Prediction (Hi-GeoMVP) model for predicting drug responses in cancer cell lines. Hi-GeoMVP combines a hierarchical drug representation module and a multi-omics integration module. The former encodes drug features through a geometry-enhanced GNN (Fang *et al.* 2022) to extract both 2D and 3D drug structures. The latter uses GNNs to learn the omics representation from omics-specific graphs incorporating gene interactions.

Beyond this, Hi-GeoMVP employs a multi-view-omics-awarded fusion module to generate final predictions by fusing high-level drug and omics representation. Lastly, the model features a regularization module that enhances drug response prediction through multi-task learning. This module, based on the premise that similar genetic profiles and drug properties may lead to similar drug responses (Wang *et al.* 2017, Zhu *et al.* 2022), involves predicting the cancer type for each cell line, the drug’s primary target pathways and drug-specific sensitivity (Iorio *et al.* 2016).

**Figure 1.**
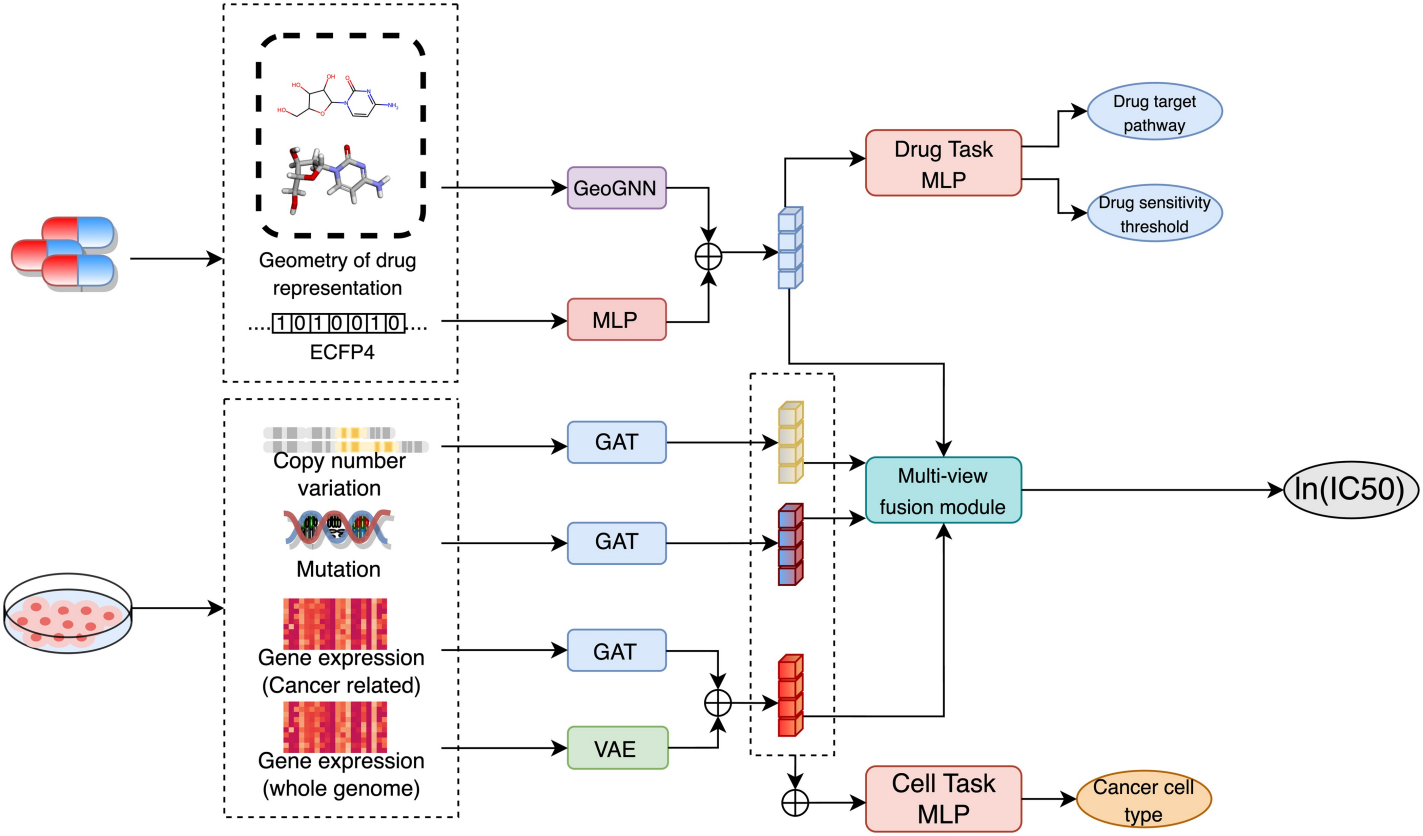
An overview of the Hi-GeoMVP model. Hi-GeoMVP takes drug geometry, chemical graph, and fingerprints, along with multi-omics data as input. Hi-GeoMVP employs a geometry-enhanced graph neural network (GeoGNN) and a multi-layer perceptron (MLP) for drug representation learning from drug features, and graph attention networks (GATs) and a variational autoencoder (VAE) for cell representation learning from multi-omics data. These representations are then integrated via a multi-view fusion module for drug response prediction (ln(IC50)). The model also utilizes latent representations alongside distinct prediction sub-networks for joint learning of drug and cell tasks, ultimately improving the prediction of drug response. The final output of the Hi-GeoMVP model is the predicted value of ln(IC50).

In a nutshell, the Hi-GeoMVP is an end-to-end DL model (Fig. 1). Key contributions of our work include:

- The introduction of the DL model that considers the 3D structure of drug representation for drug response prediction. The drug latent representation is hierarchically learned for more accurate predictions.
- The utilization of multi-task learning that incorporates auxiliary biological tasks enables robust representations for drugs and cell lines, resulting in improved predictions of drug response.
- Our model achieves state-of-the-art performance on the mix, cell blind, and drug-blind tests on the GDSC dataset.

## 2 Method

We first introduce Hi-GeoMVP by starting with the formal definition of the drug response prediction problem. Next, we present the four modules constituting Hi-GeoMVP. Lastly, we outline the training, validation, and evaluation methods for the model under various experimental conditions.

### 2.1 Drug response prediction problems

Drug response prediction is a supervised machine-learning task with the objective to derive a function that maps a drug and a cell line to the response elicited by the cell line when treated with the drug. Assuming a dataset comprising *m* drugs and *n* cell lines, the drug response profiles are represented by an n × m matrix $D=(D_{ij}$), where $D_{ij}$ denotes the response of the *i*th cell line to the *j*th drug. Drug response is quantified using the log-normalized half maximal inhibitory concentration (ln(IC50)).

The response function is learned by processing genomic profiles of the cell lines, which include gene expression, mutation, and copy number variation (CNV), as well as the drug profiles (molecular fingerprint, molecule graphs, and geometric structures).

### 2.2 The Hi-GeoMVP: geometry-enhanced drug encoder

We use GIN (Hu *et al.* 2020) to learn the drug representations from the chemical graphs. The representation for an atom at the *k*th layer, $h_{u}^{\left(k\right)}$, is updated with the message-passing scheme (Gilmer *et al.* 2017). Initially, $h_{u}^{\left(0\right)}=d^{\mathrm{atom}-u}$, where $d^{\mathrm{atom}-u}$ is the input atom attribute vector. To include the geometric relations of drugs, we employ GeoGNN (Fang *et al.* 2022), which considers the atom-to-atom (A2A) graph and the bond-to-bond (B2B) graph. Two bonds are considered connected in the B2B graph if they share a common atom. The bond representation $h_{uv}^{\left(k\right)}$ at the *k*th layer is updated by considering message passing among neighboring bonds and including the angle between connected bonds (Supplementary Fig. S1). At the final layer *K*, we apply a max pooling operation to all atoms to generate the geometric-graph level representation $h^{\mathrm{geo}}$, encoding the topological and geometric information of the drug. The detailed formulas are shown in Supplementary Document Section 1.

We utilize a multi-layer perceptron (MLP) to capture local information of a drug from its extended connectivity fingerprints (ECFP) data (Rogers and Hahn 2010), learning a representation $h^{fp}$. The concatenation of $h^{\mathrm{geo}}$ and $h^{fp}$ provides the hierarchical representation $h^{\mathrm{drug}}$ for each drug.

### 2.3 The Hi-GeoMVP: multi-view cell line encoder

We integrate gene interaction and omics features for three kinds of omics profiles: mutation, CNV, and gene expression, linked to COSMIC cancer-related genes (Tate *et al.* 2019). Each gene in the cell line graph is a node, with gene interactions forming edges and omics features acting as node attributes. For mutation and CNV profiles, cosine similarity is used to create gene interactions, while HumanNet V3’s Gold Standard Positives (GSPs) are employed for gene expression profiles (Kim *et al.* 2022). We adopted the learning approach from TGSA (Zhu *et al.* 2022), using super-nodes clustered by Graclus (Dhillon *et al.* 2007) and graph attention neural network to learn the representation for each omics data. As discussed in Zhu *et al.* (2022), Graclus only takes into account the cell line graph’s topology and not the omics features. Yet, since the cosine similarity of gene features was utilized to construct the cell line graph, the omics features were implicitly factored in during the clustering. We used the variational autoencoder (VAE) to learn an embedding from the full gene expression, which encapsulates all significant gene expression data, not merely cancer-related genes.

Finally, the multi-view cell line encoder produces multi-omics-specific hidden vectors $h^{ge}$, $h^{\mathrm{mut}}$, and $h^{\mathrm{cnv}}$, using the GAT and VAE methods. These vectors are then concatenated to form the final cell line representation, $h^{\mathrm{cell}}$, effectively integrating information from the different omics profiles.

### 2.4 The Hi-GeoMVP: omics-specific fusion layer

The omics-specific fusion layer captures drug–omics interactions individually, resulting in three pairs: gene expression and drug ($h^{ge}$, $h^{\mathrm{drug}}$), mutation and drug ($h^{\mathrm{mut}}$, $h^{\mathrm{drug}}$), and CNV and drug ($h^{\mathrm{cnv}}$, $h^{\mathrm{drug}}$). Each pair is processed through separate MLPs to produce fused drug–omics embeddings: $h^{d-ge}$, $h^{d-\mathrm{mut}}$, and $h^{d-\mathrm{cnv}}$. Each pair is processed through separate MLPs to produce fused drug–omics embeddings: $h^{d-ge}$, $h^{d-\mathrm{mut}}$, and $h^{d-\mathrm{cnv}}$. These fused embeddings are combined with the initial drug and cell vectors, resulting in a final composite vector ($h^{d-ge}$, $h^{d-\mathrm{mut}}$, $h^{d-\mathrm{cnv}}$, $h^{\mathrm{drug}}$, $h^{\mathrm{cell}}$), to make the final drug response prediction.

Our model explicitly models the drug–omics interactions. Moreover, by leveraging jumping knowledge connection, we enhance the model’s learning capability through the inclusion of $h^{\mathrm{drug}}$ and $h^{\mathrm{cell}}$s in the final prediction layer. This design preserves strong gradients and facilitates a direct information flow during training.

### 2.5 Regularization with cancer cell types, drug target pathway, and drug sensitivity threshold

We integrate biological and pharmacological knowledge into the learning process by adopting a multi-task learning approach. This approach harnesses the learned representations, $h^{\mathrm{drug}}$ and $h^{\mathrm{cell}}$, for three related tasks: classification of cell lines according to their tissue cancer types, categorization of drugs based on their targeted pathways, and prediction of drug sensitivity thresholds.

The heterogeneity of cell lines across different cancer types limits the model prediction accuracy and generalization ability (Partin *et al.* 2023). In response to this, our model is designed not only to predict pan-cancer drug responses but also to differentiate between various cancer types based on $h^{\mathrm{cell}}$.

Our model categorizes drugs by target pathways from $h^{\mathrm{drug}}$, enriching its understanding of molecular mechanisms. By employing the multi-task learning framework, our model implicitly integrates the pathway information to learn the robust drug representation $h^{\mathrm{drug}}$, proving more efficient than models that explicitly use pathway data (Li *et al.* 2023).

The drug sensitivity threshold is another important aspect we consider. We define this threshold for each drug based on its response across various cell lines (Iorio *et al.* 2016). This threshold serves as an indication of the drug’s potency in inhibiting cancer cell growth or inducing cell death.

The model learned shared representations $h^{\mathrm{drug}}$ and $h^{\mathrm{cell}}$ by optimizing the final loss function:

(1) $$L_{\text{total}}=L_{\text{pred}}+\alpha_{1}L_{\text{cancer}}+\alpha_{2}L_{\text{pathways}}+\alpha_{3}L_{\text{threshold}}$$

where $L_{\text{pred}}$ denoted the loss between the predicted drug response and the true value, $L_{\text{cancer}}$ represented the classification loss for cancer type classification of cell lines, $L_{\text{pathways}}$ was the classification loss for drug targeted pathways threshold was the regression loss to predict the drug sensitivity thresholds.

### 2.6 Model training and evaluation

For effective training of our Hi-GeoMVP model, we incorporated graph normalization (Cai *et al.* 2021) after each GNN message passing layer. To alleviate overfitting effect, we implemented an early stopping based on a separate validation set. The model was validated every five epochs, and training stopped if there was no improvement in validation error for eight consecutive iterations. We performed a grid search across the hyperparameter space given in Supplementary Table S1. The optimal set of hyperparameters was then employed to train and test the model. The detailed training step is illustrated in Supplementary Document Section 2.

As per the best practices highlighted in recent survey papers (Chen and Zhang 2022, Partin *et al.* 2023), we employed two different data split strategies for model performance evaluation:

- **Drug response prediction for known drug–cell line pairs.** We first split the entire GDSC dataset (125 696 instances) into a 80% development set and a 20% test set. Further, the development set was divided into a 90% training subset and a 10% validation subset. This procedure was repeated five times as cross-validation, and the model was then evaluated on the test set.
- **Blind test for new cell lines or drugs.** We performed a leave-drug/cell-out test, where the model predicts drug response for new drugs or cell lines unseen during training. We split the dataset by cell line and drug tin the development and test sets using the same ratio and evaluated in the same way as the first case.

The details of our evaluation are introduced in Supplementary Document Section 3. Our model’s performance was benchmarked against four other DL models: DeepCDR (Liu *et al.* 2020), TGSA (Zhu *et al.* 2022), GraphCDR (Nguyen *et al.* 2022), and NeRD (Cheng *et al.* 2022).

We assessed the model using four different metrics: the root mean square error (RMSE), mean absolute error (MAE), Pearson correlation coefficient (PCC), and $R^{2}$ statistic. The repeated results for both mix and blind tests are presented in Supplementary Figs S2–S5.

Furthermore, 10 conventional machine learning (ML) methods were compared. To specify, we evaluated five regression-based methods: ridge regression, lasso regression, elastic-net (Iorio *et al.* 2016), support vector regression (SVR) (Parca *et al.* 2019), and kernel ridge regression (KRR), three MF-based methods: CaDRRes (Suphavilai *et al.* 2018), DualNets (Zhang *et al.* 2015), and SRMF (Wang *et al.* 2017), and two miscellaneous methods: random forest, and KRR (He *et al.* 2018).

## 3 Results

In this section, we conduct a comprehensive evaluation of our proposed model, Hi-GeoMVP. Our assessment includes direct comparison with cutting-edge models, examination of the impact of our developed modules through ablation studies, and a discussion on the significant biological insights that emerged from our study.

### 3.1 The Hi-GeoMVP outperforms current methods

For a comprehensive evaluation, we compared Hi-GeoMVP with current state-of-the-art DL models, including DeepCDR, GraphDRP, TGSA, and NeRD. The DL architecture and the data used in each model are compared in Table 1. We also evaluated conventional ML approaches, with the outcomes reported in Supplementary Tables S4 and S5. The best-performing traditional ML model, SRMF, is compared with the DL methods in this section.

**Table 1.** Data usage and encoder types for DeepCDR, TGSA, GraphDRP, NeRD, and Hi-GeoMVP.

Method	Data type		Model architectures	
	Cell profiles	Drug profiles	Cell line	Drug
GraphDRP	mut	mol. graph	CNN	GIN
DeepCDR	exp + mut + meth	mol. graph	CNN + MLP	UGCN
TGSA	exp + mut + cnv + PPI	mol. graph	GAT	GIN
NeRD	miRNA + cnv	mol. graph + fingerprints	CNN + VAE	GCN + CNN
Hi-GeoMVP	exp + mut + cnv + PPI	mol. topology information	VAE + GAT	GeoGNN + MLP

Mut: mutational profile; Exp: gene expression profile; Meth: methylation profile; CNV: copy number variation profile; PPI: protein–protein interaction network; CNN: convolution neural network; GIN: graph isomorphism network; GAT: graph attention networks; GeoGNN: geometry-enhanced graph neural network; MLP: multi-layer perceptron; VAE: variational auto-encoder.

#### 3.1.1 Prediction on the known drug–cell line pairs

As evident from our study, models integrating multi-omics data generally surpassed those relying on single omics data. For instance, DeepCDR, which incorporates gene expression, mutation, and methylation data, achieved a PCC of 0.926, surpassing GraphDRP’s 0.924 and SRMF’s 0.913. DL methods outperformed SRMF, the best-performing machine learning approach, thus demonstrating their overall superiority over all traditional machine learning methods.

The Hi-GeoMVP model showed superior performance across all metrics, achieving a PCC of 0.941, $R^{2}$ of 0.880, RMSE of 0.931, and MAE of 0.680 (Table 2), underscoring the efficacy of Hi-GeoMVP in predicting drug responses for drug–cell line pairs.

**Table 2.** Performances of Hi-GeoMVP and five baseline models.

Method	PCC ↑	$R^{2}$ ↑	RMSE ↓	MAE ↓
SRMF	0.913 ± 0.0041	0.830 ± 0.0035	1.116 ± 0.0326	0.803 ± 0.0232
DeepCDR	0.926 ± 0.0026	0.832 ± 0.0090	1.110 ± 0.0298	0.864 ± 0.0309
GraphDRP	0.924 ± 0.0011	0.834 ± 0.0041	1.102 ± 0.0134	0.853 ± 0.0118
TGSA	0.928 ± 0.0008	0.861 ± 0.0015	1.009 ± 0.0053	0.744 ± 0.0053
NeRD	0.934 ± 0.0012	0.872 ± 0.0021	0.969 ± 0.0080	0.705 ± 0.0056
Hi-GeoMVP	**0.941** ± **0.0006**	**0.880** ± **0.0008**	**0.931** ± **0.0030**	**0.680** ± **0.0018**

The values displayed are the means and standard deviations from five repeated tests. The best performance is highlighted in bold. PCC: Pearson correlation coefficient; $R^{2}$: R-squared statistic; RMSE: root mean square error; MAE: mean absolute error.

**Figure 2.**
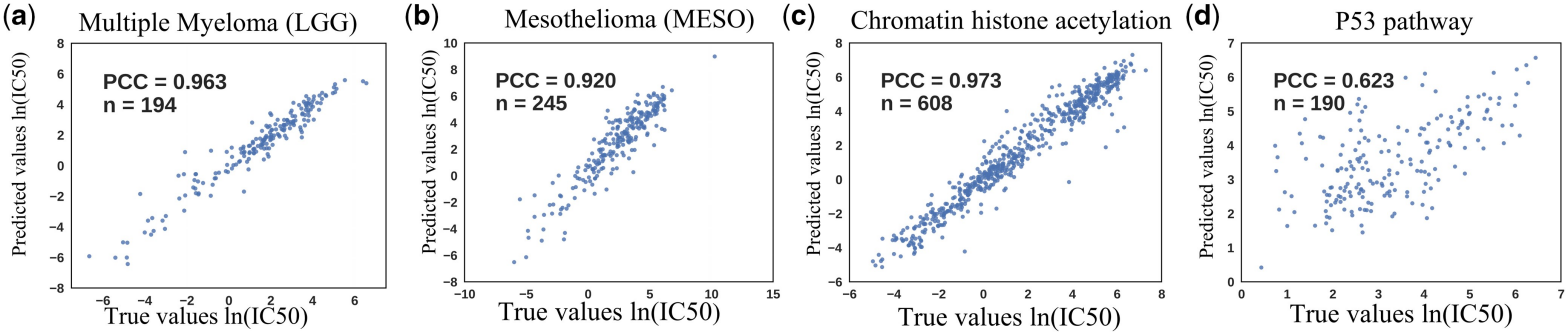
(a) and (b) Scatter plots for the best (multiple myeloma) and worst (mesothelioma) performance on cancer types in TCGA using the Hi-GeoMVP. (c) and (d) Scatter plots for the best (chromatin histone acetylation) and worst (P53 pathway) performance on drug target pathways using the Hi-GeoMVP

Our research emphasizes Hi-GeoMVP’s adaptability and accuracy in predicting drug responses across 26 varied cancer types. This understanding is key in precision medicine, as drug response variability often links to unique molecular and genetic cancer profiles, thereby necessitating bespoke therapeutic strategies (Hausser *et al.* 2019). In our analysis, we calculated the average PCCs for groups of cell lines corresponding to each cancer type. Notably, our model achieved consistently high accuracy, with PCCs ranging from 0.963 for multiple myeloma (Fig. 2a) to 0.920 for mesothelioma (Fig. 2b), and an average RMSE of 0.921 across all the cancer types (Supplementary Fig. S6). The high prediction accuracy across diverse cancer types highlights the potential of Hi-GeoMVP to advance personalized cancer treatment approaches.

**Figure 3.**
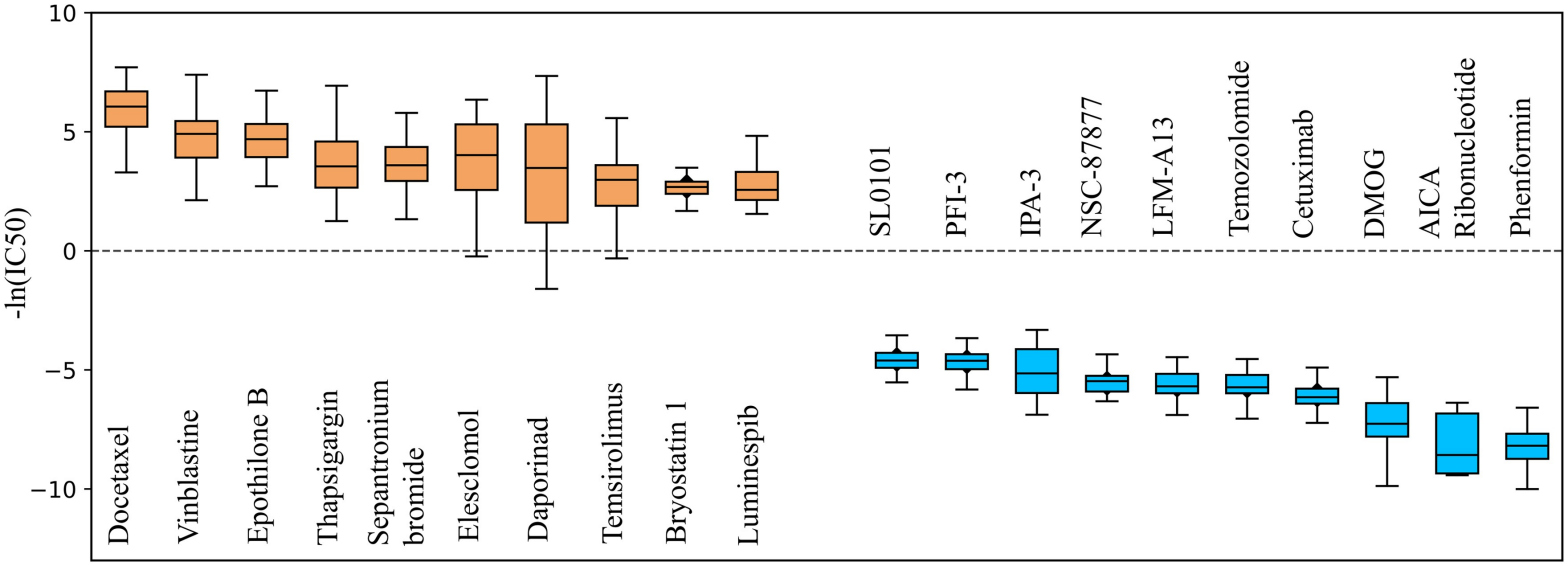
The bar plot of the mean predicted ln(IC50) for the top-five drugs with lowest (orange) and highest (blue) prediction among missing drug–cell line pairs

Evaluating the efficacy of individual drugs and grouped drugs targeting shared pathways is vital for drug development and refinement. Of the drugs examined, Belinostat, an HDAC inhibitor, yielded the best PCC of 0.964. Used in peripheral T-cell lymphoma treatment, Belinostat influences gene expression, silences oncogenes or activates tumor suppressors, thus curbing tumor growth (Tan *et al.* 2010). Further, we investigated the predictive performance of Hi-GeoMVP for drugs targeting specific biological pathways. The model showed strong predictability for the chromatin histone acetylation pathway, inclusive of Belinostat, achieving a PCC of 0.972 (Fig. 2c). This suggests Hi-GeoMVP’s potential in assisting therapeutic decision-making, especially for challenging cases where HDAC inhibitors have demonstrated promise. In contrast, drugs targeting the p53 pathway registered a lower average PCC of 0.634 (Fig. 2d).

Moreover, to evaluate our model’s robustness, we tested it with an incrementally increasing size of testing data, ranging from 10% to 70% of the entire dataset. Although a performance decline is observed in all models due to reduced training data, our model consistently outperformed others across all evaluated test set sizes (Supplementary Fig. S7).

#### 3.1.2 Cell line/drug-blind tests

To further evaluate the robustness of our model and its potential in precision medicine, we conducted cell line and drug-blind tests. The cell-blind test is a simulation where patient tumor data is excluded from the training set, and drugs are selected from a list of known compounds that have been screened on other cancer cells. Additionally, the successful performance of drug-blind tests helps drug discovery. The results of the cell-blind test and the drug-blind test are presented in Table 3 and Supplementary Tables S6 and S7.

**Table 3.** Blind test performances of Hi-GeoMVP and five baseline models for new cell lines and drugs.

	New cell line		New drugs	
Method	PCC	RMSE	PCC	RMSE
SRMF	0.769	1.805	0.369	2.805
DeepCDR	0.867	1.474	0.373	2.512
GraphDRP	0.828	1.635	0.326	2.544
TGSA	0.871	**1.337**	0.452	2.468
NeRD	0.862	1.391	0.467	2.347
Hi-GeoMVP	**0.879**	1.342	**0.596**	**2.165**

The best performance is highlighted in bold.

In the cell blind test, our Hi-GeoMVP model recorded its highest PCC at 0.879, while the TGSA model achieved its best RMSE at 1.337. While both models yielded similar results across cancer types, with mean PCCs of 0.889 for Hi-GeoMVP and 0.884 for TGSA (Supplementary Fig. S8), Hi-GeoMVP notably excelled in per-drug results, showing a significantly higher mean PCC of 0.358 against TGSA’s 0.312 (*P*-value: $4\times 10^{-4}$, Supplementary Fig. S9).

The drug-blind test is harder than the cell-blind test due to the vast chemical space of drugs, making it difficult to learn generalizable embedding with limited screened drugs (Partin *et al.* 2023). Among the competing models, NeRD and Hi-GeoMVP, both utilizing drug FPs and drug chemical graphs, stood out. Hi-GeoMVP, leveraging drug geometry, achieved a PCC of 0.596, a 27.6% increase over NeRD’s 0.467, and emerged as the top performer across all metrics.

### 3.2 Hi-GeoMVP facilitates biological findings

Beyond quantitative performance metrics, we validated the biological relevance of our model’s predictions. It provides an alternative validation of the model’s effectiveness and also demonstrates its potential to generate new biological insights.

#### 3.2.1 The Hi-GeoMVP discovers unknown drug responses

We trained Hi-GeoMVP on the known drug–cell line pairs and made predictions for 4222 undocumented instances. One interesting prediction involved the drug–cell line pair Daporinad and SUP-B15, which had the lowest predicted ln(IC50) value (−8.15) (see Fig. 3). Daporinad inhibits NAMPT, an enzyme whose suppression has been found effective in hindering leukemia progression in B-ALL cell lines like SUP-B15, aligning with our prediction (Takao *et al.* 2018).

#### 3.2.2 The Hi-GeoMVP identifies drug-targeted pathways

Predicting drug-targeted signaling pathways is vital in drug discovery. Hi-GeoMVP predicted CCT007093 targeting the p53 pathway, aligning with established literature (Rayter *et al.* 2008). Our model also correctly predicted kinase inhibitors GSK650394 and NVP-BHG712, targeting SGK1 kinase and EphB4 kinase (Martiny-Baron *et al.* 2010) respectively, even though these drugs are listed under ‘other’.

### 3.3 Ablation study

To substantiate the advantages of Hi-GeoMVP, we delve into an ablation study. The study shows that the use of hierarchical features of the drugs plays a vital role in the performance of the model (Table 4). The inclusion of the drug’s chemical graph alone leads to a PCC of 0.927. However, the combination of the chemical graph and fingerprint of drugs, the PCC increased to 0.932. The utility of drug geometry information [Hi-GeoMVP (no regularization)] further improves the PCC to 0.937. This underscores the importance of feature combinations in achieving superior model performance.

**Table 4.** Ablation studies for Hi-GeoMVP under different settings.

Experiment setting	PCC
Only drug’s chemical graph	0.927
Drug’s chemical graph + FP	0.932
Hi-GeoMVP (no regularization)	0.937
Hi-GeoMVP (cancer-regular only)	0.939
Hi-GeoMVP (pathway-regular only)	0.939
Hi-GeoMVP	0.941

Next, we investigate the impact of multi-task learning. We compare the model’s performance when predicting drug response alone [Hi-GeoMVP (no regularization)], versus when incorporating additional tasks related to cancer types and pathway activities [Hi-GeoMVP (cancer-regular only), Hi-GeoMVP (pathway-regular only), and Hi-GeoMVP]. The results show that the introduction of multi-task learning leads to a slight increase in PCC from 0.937 to 0.939. When both auxiliary tasks are included (Hi-GeoMVP), the PCC improves further to 0.941.

## 4 Datasets and data preparation

We employed the GDSC datasets, containing 125 696 drug–cell line pairs with 177 drugs and 734 cancer cell lines.

For cell line profiles, we integrated gene expression, CNV, and mutation data from the GDSC dataset. We removed genes with minimal expression variation yielding a whole gene expression set ($c^{ge-\mathrm{whole}}\in R^{734\times 8046}$).

Subsequently, we refined the data by filtering for genes specific to cancer as listed in the COSMIC dataset. The final dataset includes the complete gene expression data ($c^{ge-\mathrm{whole}}\in R^{734\times 8046}$), filtered gene expression data ($c^{ge}\in R^{734\times 414}$), mutation data ($c^{\mathrm{mut}}\in {\left\{0,1\right\}}^{734\times 636}$), and CNV data ($c^{\mathrm{cnv}}\in {\left\{0,1\right\}}^{734\times 696}$).

We further constructed cell line graphs for each genomic data type. For gene expression, we utilized experimentally validated gene pairs from HumanNet (Kim *et al.* 2022) to infer gene interactions. For mutation and CNV data, we constructed graphs based on cosine similarity scores due to the limited number of genes aligning with GSPs in these omics sets.

For a cancer drug $d_{j}$, we formulated chemical graphs where each atom was represented as a node and each bond as an edge. We used seven distinct atom features and three bond features (Supplementary Tables S2 and S3 for more details) to establish an atom feature matrix ($d_{j}^{\mathrm{atom}}$) and a bond feature matrix ($d_{j}^{\mathrm{bond}}$). The connectivity between the atoms was expressed through the adjacency matrix ($G_{j}^{d-\mathrm{atom}}$). We included geometric information about the drug by calculating the angles between bonds, which led to a B2B connection alongside the A2A connection. Consequently, this atom–bond–angle relation embraces both the topology and the geometry of the drugs (Fang *et al.* 2022). Finally, we utilized the ECFPs (Rogers and Hahn 2010) to enhance the prediction accuracy through the integration of the hierarchical representation of the drug. The drug and cell profiles are detailed in Supplementary Document Section 4.

## 5 Discussion

In this work, we introduced Hi-GeoMVP for accurate prediction of drug responses in cancer cell lines. It incorporates a hierarchical set of drug features with three-dimensional geometry, leading to notably enhanced performance. It achieves the state-of-the-art performance in both mixed and blind tests, highlighting its potential in personalized cancer treatment. We propose three future directions that could enhance our model.

Firstly, we have demonstrated the advantages of incorporating multi-task learning as a regularization term. Additionally, our design can accommodate other auxiliary tasks derived from biological and pharmacological knowledge related to drug response prediction, such as drug target prediction (Pak *et al.* 2023) and gene–disease association prediction. Investigating different combinations of these tasks may further refine drug response predictions and contribute to understanding biological mechanisms.

Secondly, our current handling of multi-omics data involves using separate encoders for each type of omics data and filtering cell lines with incomplete or differing missing data. Recent studies have proposed tackling this ‘missing type’ issue by modeling joint representations as products of marginal representations derived from observed omics data with different missing patterns (Lee and van der Schaar 2021). Therefore, developing a more adaptable cell line encoder becomes an imperative step in our future work for drug response prediction.

Lastly, TGSA achieved comparable performance with our model in cell blind test, which can be attributed to both employing cell line GNN on cancer-related genes. Hi-GeoMVP further incorporates the complete gene sets by using VAE. Exploring gene–gene interactions within the full gene spectrum, rather than limiting to cancer-specific genes, could yield more accurate results, especially with a scalable GNN model.

## Supplementary Material

btae204_Supplementary_Data

## Data Availability

The GDSC drug response data is publicly available at https://www.cancerrxgene.org/downloads/bulk_download. The processed multi-omics features can be downloaded from the https://github.com/Jinyu2019/Suppl-data-BBpaper. The protein-protein interaction is publicly available at https://staging2.inetbio.org/humannetv3/download.php. The code is public and available on GitHub at https://github.com/matcyr/Hi-GeoMVP.

## References

[btae204-B1] Barretina J , Caponigro G, Stransky N et al The cancer cell line encyclopedia enables predictive modelling of anticancer drug sensitivity. Nature 2012;483:603–7. 22460905 10.1038/nature11003 PMC3320027

[btae204-B2] Cai T , Luo S, Xu K et al Graphnorm: a principled approach to accelerating graph neural network training. In: *ICML*, Vienna, Austria, PMLR, 2021, vol. 139, pp. 1204–1215.

[btae204-B3] Chapman PB , Hauschild A, Robert C, et al; BRIM-3 Study Group. Improved survival with vemurafenib in melanoma with BRAF V600E mutation. N Engl J Med 2011;364:2507–16. 21639808 10.1056/NEJMoa1103782 PMC3549296

[btae204-B4] Chen J , Zhang L. A survey and systematic assessment of computational methods for drug response prediction. Brief Bioinform 2021;22:232–46. 31927568 10.1093/bib/bbz164

[btae204-B5] Chen Y , Zhang L. How much can deep learning improve prediction of the responses to drugs in cancer cell lines? Brief Bioinform 2022;23:bbab378. 34529029 10.1093/bib/bbab378

[btae204-B6] Cheng X , Dai C, Wen Y et al NeRD: a multichannel neural network to predict cellular response of drugs by integrating multidimensional data. BMC Med 2022;20:368. 36244991 10.1186/s12916-022-02549-0 PMC9575288

[btae204-B7] Dasari S , Tchounwou PB. Cisplatin in cancer therapy: molecular mechanisms of action. Eur J Pharmacol 2014;740:364–78. 25058905 10.1016/j.ejphar.2014.07.025 PMC4146684

[btae204-B8] Dhillon IS , Guan Y, Kulis B. Weighted graph cuts without eigenvectors a multilevel approach. IEEE Trans Pattern Anal Mach Intell 2007;29:1944–57. 17848776 10.1109/TPAMI.2007.1115

[btae204-B9] Fang X , Liu L, Lei J et al Geometry-enhanced molecular representation learning for property prediction. Nat Mach Intell 2022;4:127–34.

[btae204-B10] Gillet JP , Varma S, Gottesman MM. The clinical relevance of cancer cell lines. J Natl Cancer Inst 2013;105:452–8. 23434901 10.1093/jnci/djt007 PMC3691946

[btae204-B11] Gilmer J , Schoenholz S, Riley P et al Neural message passing for quantum chemistry. In: *ICML, Sydney, Australia*., PMLR, 2017, vol. 70, pp. 1263–1272.

[btae204-B12] Hausser J , Szekely P, Bar N et al Tumor diversity and the trade-off between universal cancer tasks. Nat Commun 2019;10:5423. 31780652 10.1038/s41467-019-13195-1 PMC6882839

[btae204-B13] He X , Folkman L, Borgwardt K. Kernelized rank learning for personalized drug recommendation. Bioinformatics 2018;34:2808–16. 29528376 10.1093/bioinformatics/bty132 PMC6084606

[btae204-B14] Hu W , Liu B, Gomes J et al. Strategies for pre-training graph neural networks. In: *ICLR*, Addis Ababa, Ethiopia, 2020.

[btae204-B15] Iorio F , Knijnenburg TA, Vis DJ et al A landscape of pharmacogenomic interactions in cancer. Cell 2016;166:740–54. 27397505 10.1016/j.cell.2016.06.017 PMC4967469

[btae204-B16] Kim CY , Baek S, Cha J et al HumanNet v3: an improved database of human gene networks for disease research. Nucleic Acids Res 2022;50:D632–D639. 34747468 10.1093/nar/gkab1048 PMC8728227

[btae204-B17] Lee C , van der Schaar M. A variational information bottleneck approach to multi-omics data integration. In: AISTATS, Virtual Event, PMLR, 2021, vol. 130, pp. 1513–1521.

[btae204-B18] Li Y , Hostallero DE, Emad A. Interpretable deep learning architectures for improving drug response prediction performance: myth or reality? Bioinformatics 2023;39(6):btad390. 10.1093/bioinformatics/btad390 PMC10301685 37326960

[btae204-B19] Liu Q , Hu Z, Jiang R et al DeepCDR: a hybrid graph convolutional network for predicting cancer drug response. Bioinformatics 2020;36:i911–18. 33381841 10.1093/bioinformatics/btaa822

[btae204-B20] Martiny-Baron G , Holzer P, Billy E et al The small molecule specific EphB4 kinase inhibitor NVP-BHG712 inhibits VEGF driven angiogenesis. Angiogenesis 2010;13:259–67. 20803239 10.1007/s10456-010-9183-z PMC2941628

[btae204-B21] Nguyen T , Nguyen GTT, Nguyen T et al Graph convolutional networks for drug response prediction. IEEE/ACM Trans Comput Biol Bioinform 2022;19:146–54. 33606633 10.1109/TCBB.2021.3060430

[btae204-B22] Pak M , Lee S, Sung I et al Improved drug response prediction by drug target data integration via Network-Based profiling. Brief. Bioinform 2023;24:bbad034. 36752352 10.1093/bib/bbad034

[btae204-B23] Parca L , Pepe G, Pietrosanto M et al Modeling cancer drug response through drug-specific informative genes. Sci Rep 2019;9:15222. 31645597 10.1038/s41598-019-50720-0 PMC6811538

[btae204-B24] Partin A , Brettin TS, Zhu Y et al Deep learning methods for drug response prediction in cancer: predominant and emerging trends. Front Med (Lausanne) 2023;10:1086097. 36873878 10.3389/fmed.2023.1086097 PMC9975164

[btae204-B25] Rayter S , Elliott R, Travers J et al A chemical inhibitor of PPM1D that selectively kills cells overexpressing PPM1D. Oncogene 2008;27:1036–44. 17700519 10.1038/sj.onc.1210729

[btae204-B26] Rogers D , Hahn M. Extended-connectivity fingerprints. J Chem Inf Model 2010;50:742–54. 20426451 10.1021/ci100050t

[btae204-B27] Shen B , Feng F, Li K et al. A systematic assessment of deep learning methods for drug response prediction: from in vitro to clinical applications. Brief Bioinform 2023;24(1):bbac605. 10.1093/bib/bbac605 36575826

[btae204-B28] Suphavilai C , Bertrand D, Nagarajan N et al Predicting cancer drug response using a recommender system. Bioinformatics 2018;34:3907–14. 29868820 10.1093/bioinformatics/bty452

[btae204-B29] Takao S , Chien W, Madan V et al Targeting the vulnerability to NAD+ depletion in B-cell acute lymphoblastic leukemia. Leukemia 2018;32:616–25. 28904384 10.1038/leu.2017.281

[btae204-B30] Tan J , Cang S, Ma Y et al Novel histone deacetylase inhibitors in clinical trials as anti-cancer agents. J Hematol Oncol 2010;3:5. 20132536 10.1186/1756-8722-3-5 PMC2827364

[btae204-B31] Tate JG , Bamford S, Jubb HC et al COSMIC: the catalogue of somatic mutations in cancer. Nucleic Acids Res 2019;47:D941–47. 30371878 10.1093/nar/gky1015 PMC6323903

[btae204-B32] Veličković P, Cucurull G, Casanova A et al Graph attention networks. In: *ICLR*, Vancouver, BC, Canada, 2018.

[btae204-B33] Wang D , Hensman J, Kutkaite G et al, GDSC Screening Team. A statistical framework for assessing pharmacological responses and biomarkers using uncertainty estimates. Elife 2020;9. 10.7554/eLife.60352 PMC7746236 33274713

[btae204-B34] Wang L , Li X, Zhang L et al Improved anticancer drug response prediction in cell lines using matrix factorization with similarity regularization. BMC Cancer 2017;17:513. 28768489 10.1186/s12885-017-3500-5 PMC5541434

[btae204-B35] Weinstein JN. Cell lines battle cancer. Nature 2012;483:544–5. 22460893 10.1038/483544a

[btae204-B36] Zhang N , Wang H, Fang Y et al Predicting anticancer drug responses using a dual-layer integrated cell line-drug network model. PLoS Comput Biol 2015;11:e1004498. 26418249 10.1371/journal.pcbi.1004498 PMC4587957

[btae204-B37] Zhu Y , Ouyang Z, Chen W et al TGSA: protein-protein association-based twin graph neural networks for drug response prediction with similarity augmentation. Bioinformatics 2022;38:461–8. 34559177 10.1093/bioinformatics/btab650

