# Supplementary material for: Hi-GeoMVP: a hierarchical geometry-enhanced deep learning model for drug response prediction
Source: Bioinformatics. 2024 Apr 13;40(4):btae204. doi: 10.1093/bioinformatics/btae204 (PMC11060866; doi:10.1093/bioinformatics/btae204)
Supplement: btae204_Supplementary_Data [file btae204_supplementary_data.pdf]

Supplementary Document

Yurui Chen, and Louxin Zhang

February 4, 2024

1 Deep learning backbone

We used graph isomorphism networks [1], graph attention networks [2], and the variational autoencoder [3] as the backbone to learn the drug and cell line representations respectively.

1.1 Drug encoders

Graph Isomorphism Network

Graph isomorphism network (GIN) is a message-passing graph neural network [4]. Each node feature is recursively from the messages of its neighbor nodes at each layer. The k-th layer GIN cooperating with bond information is:

$$h_u^{(k)} = \text{MLP}^{(k)}\left(h_u^{(k-1)} + \sum_{v\in\mathcal{N}(u)} \text{ReLU}\left(h_v^{(k-1)} + d^{bond-uv}\right)\right) \quad (1)$$

where MLP^k is the Multilayer Perceptron at k-th layer that maps the aggregated messages for u to the drug latent space. Here, $h_u^{(k)}$ denotes the representation for atom u at the k-th layer, $d^{bond-uv}$ is the initial bond attribute vector for bond uv, $\mathcal{N}(u)$ is the set of neighboring atoms of atom u, and $h_u^{(0)} = d^{atom-u}$.

Geometry-enhanced Graph Neural Network

We adopt the GeoGNN [5] to incorporate the drug's atom-bond-angle geometry relations. With the initialization of $h_{uv}^{(0)} = d^{bond-uv}$, the bond uv's representation $h_{uv}^{(k)}$ at the k-th layer is updated by:

$$h_{uv}^{(k)} = \text{MLP}^{(k)}\left(h_{uv}^{(k-1)} + \sum_{w\in\mathcal{N}(v)\backslash u} \text{ReLU}\left(h_{vw}^{(k-1)} + d^{angle-uvw}\right) + \sum_{w\in\mathcal{N}(u)\backslash v} \text{ReLU}\left(h_{uw}^{(k-1)} + d^{angle-wuv}\right)\right) \quad (2)$$

After the bond representation is updated at layer k, $h_u^{(k)}$ is reformulated by:

$$h_u^{(k)} = \text{MLP}^{(k)}\left(h_u^{(k-1)} + \sum_{v\in\mathcal{N}(u)} \text{ReLU}\left(h_v^{(k-1)} + h_{uv}^{(k-1)}\right)\right) \quad (3)$$

1.2 Cell encoders

Graph Attention Network

Graph attention networks apply both the attention mechanism and graph neural networks to learn the graph representation. The updating process is as follows:

- Compute attention coefficient: The attention coefficient is given by the equation:

$$e_{ij} = \text{LeakyReLU}(\mathbf{a}^T[\mathbf{W}\mathbf{h}_i||\mathbf{W}\mathbf{h}_j]) \quad (4)$$

Then, the coefficients are normalized using the softmax function so they can be considered as probabilities, allowing the model to focus on important nodes:

$$\alpha_{ij} = \text{softmax}_j(e_{ij}) = \frac{\exp(e_{ij})}{\sum_{k \in \mathcal{N}(i)} \exp(e_{ik})} \tag{5}$$

- Update the node features: The output features of the GAT are computed as a weighted sum of the features of every node, where the weights are the attention coefficients:

$$\mathbf{h}_i' = \sum_{j \in \mathcal{N}(i)} \alpha_{ij} \mathbf{W} \mathbf{h}_j \tag{6}$$

Here, $\mathbf{h}_i$ is the feature vector of the node i, $\mathbf{W}$ is a weight matrix, $[\mathbf{W}\mathbf{h}_i || \mathbf{W}\mathbf{h}_j]$ represents the concatenation of the transformed feature vectors of nodes i and j, $\mathbf{a}$ is a weight vector, LeakyReLU is the activation function, $\mathcal{N}(i)$ is the set of neighbor nodes of node i, and α_{ij} is the attention coefficient between nodes i and j.

Variational Autoencoder

We use a Variational Autoencoder (VAE) to learn the complete gene expression embedding but not only cancer-related gene expression features. VAE is a type of generative model that learns a distribution but not distinct points in the latent space. The VAE is composed of two main components: an encoder and a decoder. In the VAE model, the whole gene expression (WG) data for a given cell i, denoted as c_i^{wg}, is generated based on a latent variable z_i, which follows a prior distribution $p_\theta(z_i)$, through a conditional distribution (decoder) $p_\theta(c_i^{wg} \mid z_i)$. Variational distribution $q_\phi(z_i \mid c^{wg})$(encoder) is introduced to approximate the true posterior $p_\theta(z_i \mid c_i^{wg})$, which maps $c^{wg}i$ to a latent space. We use a $4-layer$ MLP for the encoder and the decoder. We denote the reconstructed gene expression profiles by $c^{wg'}$. The VAE updates the encoder and decoder jointly by maximizing the evidence lower bound (ELBO):

$$L_{\text{VAE}} = \mathbb{E}_{q_\phi(z|c^{wg})}\left[\log p_\theta(c^{wg} \mid z)\right] - D_{\text{KL}}\left(q_\phi(z \mid c^{wg}) | p_\theta(z)\right) \tag{7}$$

where D_{KL} is the Kullback-Leibler (KL) divergence between two probability distributions and the first term in Eq.7 is the reconstruction loss which is measured by the negative Mean Squared Error(MSE): $-MSE(c^{wg}, c^{wg'})$.

2 Model training details

We built the model using the deep learning library PyTorch, and PyTorch Geometric. We did an early stop on the validation set. The model was validated every five epochs, and training was halted if there was no improvement in validation error for eight consecutive iterations. We did the hyperparameters search on the validation set to select the best hyperparameter set. The hyperparameters are listed in Supplementary Table 1. The conventional machine learning methods are trained in Matlab (SRMF), R (DualNets), and Python (all other methods).

3 Assessment methods

We performed an assessment of various deep learning models using a shared dataset, GDSC V1, adhering to a baseline testing methodology. This drug response dataset was divided into two segments in a 2:8 ratio. The larger portion was utilized for both training and validating the neural network model, while the smaller portion was employed to measure the model's accuracy across three separate metrics.

The process of training and validating a model necessitated a further division of the larger dataset into five equally sized subsets. In each iteration, we combined four of these subsets to form the training set, leaving the fifth for validation purposes. This cycle was repeated five times, each time swith a different subset serving as the validation set.

Each experiment concluded with the calculation of the accuracy of the testing set. Ultimately, the average accuracy obtained from the five trials was recorded and reported as the final result.

We evaluated the methods using four different metrics, including Root Mean Square Error (RMSE), Pearson Correlation Coefficient (PCC), and R^2 statistic.

The root mean square error (RMSE) and mean absolute error (MAE) captures the deviation between the true and predicated responses for a method and are defined as:

$$\mathrm{RMSE}(\mathbf{y}, \widehat{\mathbf{y}}) = \sqrt{\frac{\sum_i (y_i - \widehat{y}_i)^2}{n}}, \quad (8)$$

$$\mathrm{MAE}(\mathbf{y}, \widehat{\mathbf{y}}) = \frac{\sum_i |y_i - \widehat{y}_i|}{n}. \quad (9)$$

The Pearson correlation coefficient (PCC) of $\mathbf{y}$ and $\widehat{\mathbf{y}}$ is defined as:

$$\mathrm{PCC}(\mathbf{y}, \widehat{\mathbf{y}}) = \frac{\sum_i \left(\widehat{y}_i - \mu(\widehat{\mathbf{y}})\right)\left(y_i - \mu(\mathbf{y})\right)}{\sqrt{\sum_i \left(\widehat{y}_i - \mu(\widehat{\mathbf{y}})\right)^2}\sqrt{\sum_i \left(y_i - \mu(\mathbf{y})\right)^2}}, \quad (10)$$

where the $\mu()$ represents the mean of the components of a vector.

The R squared statistic (R^2) is defined as:

$$R^2(\mathbf{y}, \widehat{\mathbf{y}}) = 1 - \frac{\sum_i (y_i - \widehat{y}_i)^2}{\sum_i \left(y_i - \mu(\mathbf{y})\right)^2} \quad (11)$$

The smaller values for RMSE and MAE indicate better performance of a method. This means the predicted responses are closer to the true values. Conversely, a value close to one for the PCC and R^2 metrics indicates good performance.

4 Hard split for blind test

In the problem of drug response prediction, models are developed based on the observation that drugs with similar chemical characteristics typically exhibit similar responses [6]. Similarly, the response of two cell lines to a particular drug tends to be alike if their genomic profiles are comparable. To rigorously assess model performance in blind tests, we implemented a more strict data splitting strategy. For the drug blind test, we divided the drugs by their scaffold structures [7], which provide a more realistic estimation of model performance in prospective evaluations compared to random splitting [8]. For the cell line blind test, we employed maximum dissimilarity sampling, utilizing cosine similarity measures of gene expression to select training, validation, and test cell lines.

5 Datasets and data preprocessing

5.1 Cell lines profiles

We adopted the gene expression, CNV, and mutation profiles presented in the GDSC dataset as the cell line features for this study. We used the same preprocessed genomic profiles in [9] where genes with low expression in variation are removed and only cancer-related gene sets according to COSMIC are left for mutation and CNV data. We further filtered the gene expression by the COSMIC cancer-related gene sets to build the gene-gene interaction graph. For multi-omics data, we got $c^{ge-whole} \in \mathbb{R}^{734\times8046}$,$c^{ge} \in \mathbb{R}^{734\times414}$,$c^{mut} \in \{0,1\}^{734\times636}$ and $c^{cnv} \in \{0,1\}^{734\times696}$. All the processed data can be downloaded from https://github.com/Jinyu2019/Suppl-data-BBpaper.

We constructed multiple cell line graphs for different genomic data. Compared to the neutral graph structure of molecular drugs, cell lines have no explicit relational structures among genes. We used the Gold

Standard Positives(GSPs) from HumanNet[10] to derive the gene interactions $G^{ge}_{GSP} \in \mathbb{R}^{414\times414}$ in our study. GSPs are experimentally validated gene pairs, making the resulting interactions more robust and reliable than those derived from PPIs. Additionally, the smaller number of interactions guarantees that the cell line graph is sparse without the need of setting any threshold for the interaction score.

Furthermore, existing studies research on GNN demonstrates that beyond the known topology, the similarity between initial node attributes also has a significant impact [11], suggesting we could consider gene interactions in a higher-level latent space. We created $G^{ge}_{sim} \in \{0,1\}^{414\times414}$ by defining gene-gene interaction using a Pearson correlation coefficient (PCC) threshold of 0.5 and a corresponding P-value threshold of 0.05 from c^{ge}. $G^{mut} \in \{0,1\}^{636\times636}$ and $G^{cnv} \in \{0,1\}^{694\times694}$ were created from c^{mut} and c^{cnv} by calculating the cosine similarity score, with a threshold set to the highest 1% similarity score. We only use a similarity-based graph for mutation and CNV since the matched genes in these two omics set with the GSPs are rather limited which may filter the important cancer-related genes.

5.2 Drug profiles

We built the 2-D chemical graph for a drug from the drug's SMILES representation using a Python package called RDKit [12]. Suppose j-th drug contains n_j atoms and m_j bonds. We denote the atom feature matrix as d^{atom}_j with k-dimensional features where $d^{atom}_j \in \mathbb{R}^{n_j\times k}$ and the bond feature matrix with l-dimensional features as $d^{bond}_j \in \mathbb{R}^{m_j\times l}$. The way how the atoms are connected is given by the adjacency matrix $G^{d-atom}_j \in \{0,1\}^{n_j\times n_j}$.

Beyond the 2-D atom-atom connection ($a2a$), we introduce the bond-bond ($b2b$) connection by considering the angle between two bonds [5]. We used the Merck Molecular Force Field (MMFF) in RDKit to generate multiple 3D conformers for a given drug and optimize their geometries to get the atom coordinates. By estimating the coordinations of each atom in the spatial domain and building the bond-bond-connection-based graph $G^{d-bond}_j \in \{0,1\}^{m_j\times m_j}$ and a bond-bond angle vector d^{angle}_j, the geometry information of the drug is captured.

6 Supplementary tables and figures

Supplementary Table S1: The possible and best hyperparameters.

**Hyper-parameters**	**Range**	**Optimal values**
Batch size	512, 256	512
Learning rate	1e-4, 5e-4, 1e-3	1e-4
GeoGNN layer number	3	3
GeoGNN hidden dim	128, 256, 512	256
GAT hidden dim	128, 256, 512	128
Multi-view dim	128, 256, 512	256
Cancer type penalty	0.5, 1	0.5
Pathway penalty	0.5, 1	0.5
Drug threshold penalty	0.5, 1	0.5

Supplementary Table S2: Atom features

**Feature type**	**# Possible Values**
Atom type	43
Chirality	5
Degree	12
Formal charge	12
Hybridization	6
Aromatic	2
Ring	2

Supplementary Table S3: Bond features

**Feature type**	**# Possible Values**
Bond type	5
Bond stereo	6
Conjugated	2

Method	PCC ↑	R^2 ↑	RMSE ↓	MAE ↓
Ridge Regression	0.847	0.717	1.439	1.076
Lasso Regression	0.849	0.720	1.430	1.072
Elastic-Net	0.850	0.722	1.426	1.068
SVR_{linear}	0.819	0.662	1.573	1.180
SVR_{poly}	0.844	0.710	1.455	1.073
SVR_{rbf}	0.846	0.716	1.442	1.067
KRR	0.849	0.721	1.429	1.071
CaDRRes	0.856	0.716	1.421	1.075
DualNets	0.862	0.733	1.396	1.005
**SRMF**	**0.913**	**0.830**	**1.116**	**0.803**
Random Forest	0.841	0.706	1.466	1.100
**Hi-GeoMVP**	**0.941**	**0.880**	**0.931**	**0.680**

Supplementary Table S4: Regression performances of machine learning models for mix tests.

Method	PCC	RMSE
Elastic-Net	0.125	2.355
SVR_{linear}	0.169	2.455
SVR_{rbf}	0.462	2.105
KRR	0.849	0.721
CaDRRes	0.688	2.015
DualNets	0.705	1.956
**SRMF**	**0.769**	**1.805**
Random Forest	0.668	1.995
**Hi-GeoMVP**	**0.879**	**1.342**

Supplementary Table S5: Regression performances of machine learning models for cell blind tests.

Supplementary Table S6: Cell Blind Test Results by Dis-similarity Split. The response of cell lines not included in the training set was predicted. The cell lines are split by the dis-similarity of gene expression. The values represent the mean of five trials, and the best performance for each metric is highlighted in bold.

Method	PCC	RMSE
DeepCDR	0.867	1.483
GraphDRP	0.847	1.621
TGSA	0.869	1.382
NeRD	0.861	1.402
Hi-GeoMVP	**0.873**	**1.352**

Supplementary Table S7: Drug Blind Test Results by Scaffolds Split. The response of drugs not included in the training set was predicted. The drugs are split by scaffold structures. The values represent the mean of five trials, and the best performance for each metric is highlighted in bold.

Method	PCC	RMSE
DeepCDR	0.278	2.866
GraphDRP	0.386	2.818
TGSA	0.307	2.772
NeRD	0.326	2.655
Hi-GeoMVP	**0.493**	**2.423**

Supplementary Figure S1: GeoGNN model architecture. LN: layer normalization.GN: Graph normalization. GIN: Graph isomorphism network.

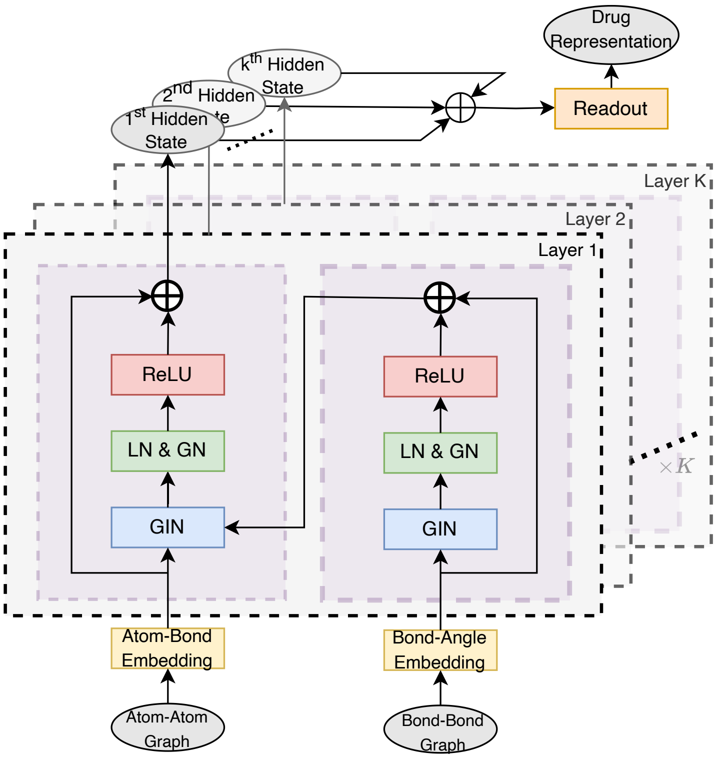

Supplementary Figure S2: Performance of Hi-GeoMVP is compared with other models in mix test for 5 repeated experiments. **(a)**, **(b)**, **(c)**, and **(d)** are results for PCC, R^2, MAE, and RMSE respectively.

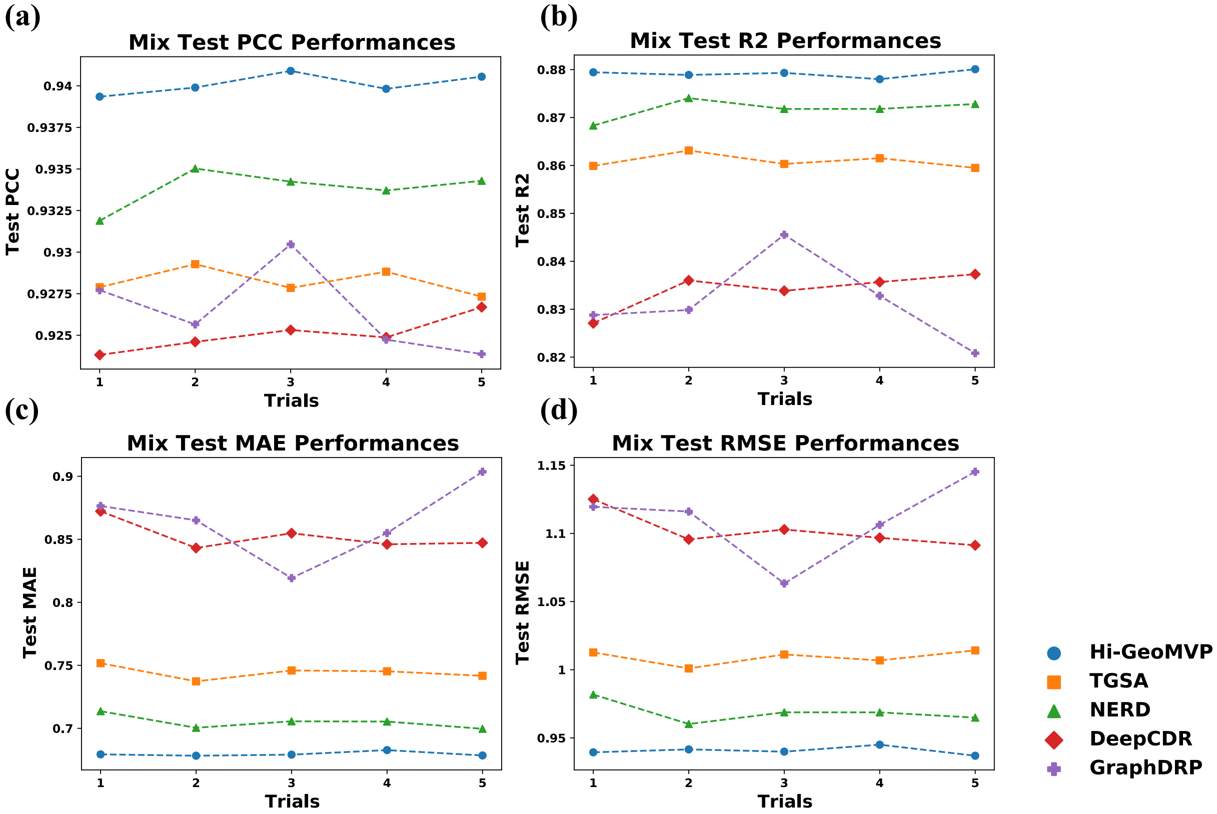

Supplementary Figure S3: Performance of Hi-GeoMVP is compared with other models in Leave-Cell line-Out test for 5 repeated experiments. **(a)**, **(b)**, **(c)**, and **(d)** are results for PCC, R^2, MAE, and RMSE respectively.

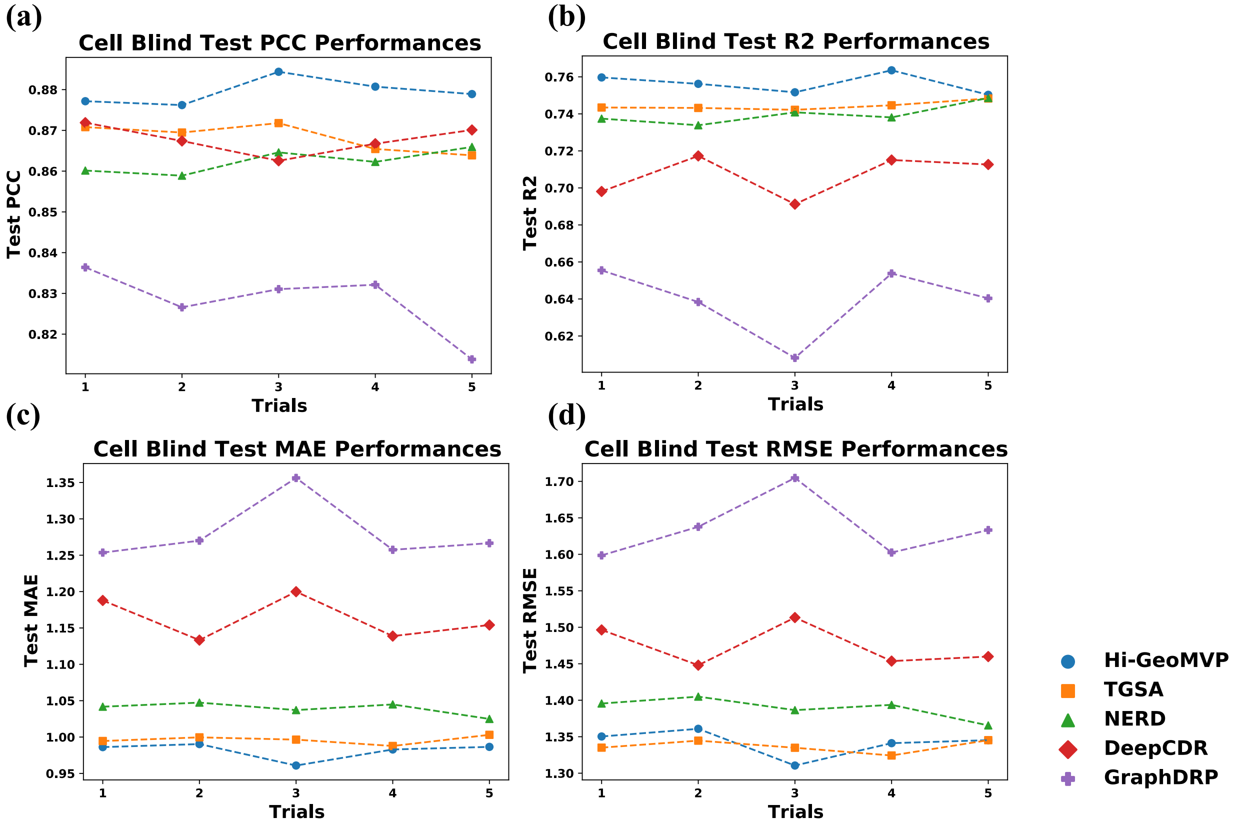

Supplementary Figure S4: Performance of Hi-GeoMVP is compared with other models in Leave-Drug-Out test for 5 repeated experiments. **(a)**, **(b)**, **(c)**, and **(d)** are results for PCC, R^2, MAE, and RMSE respectively.

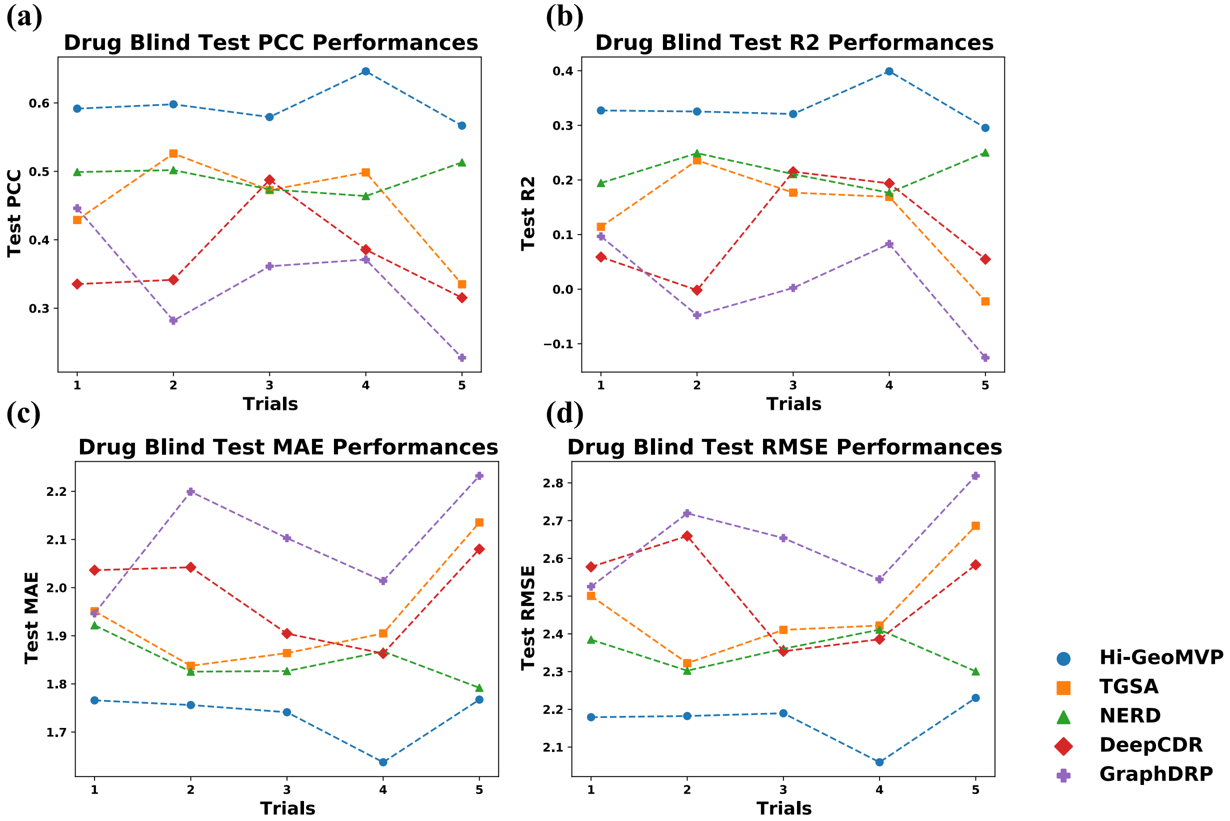

Supplementary Figure S5: Performance of Hi-GeoMVP is compared with other models in hard blind test for 5 repeated experiments. **(a)** is the test PCC for Scaffold Leave-Drug-Out test. **(a)** is the test PCC for cell-dissimilarity Leave-Cell line-Out test.

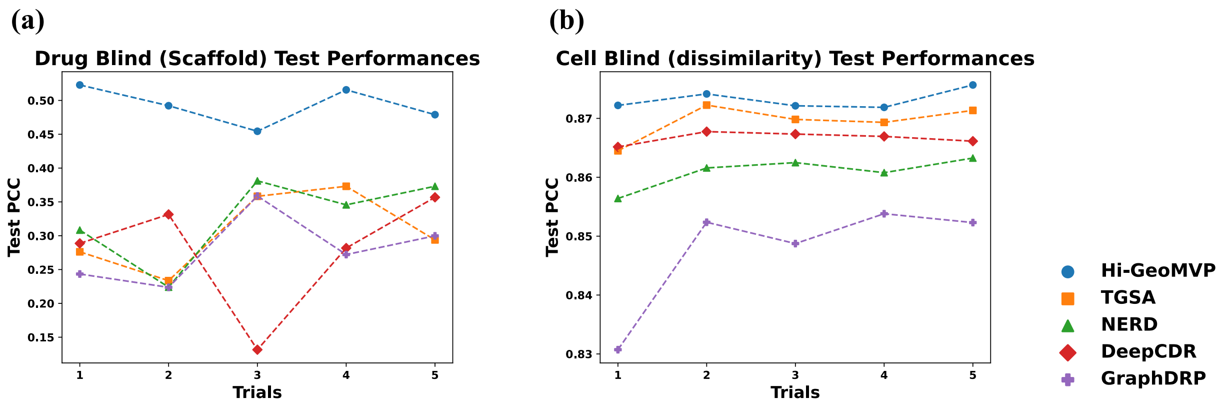

Supplementary Figure S6: **(a)** and **(b)** illustrates the distribution of RMSE per cancer type and per drug in the mixed test set.

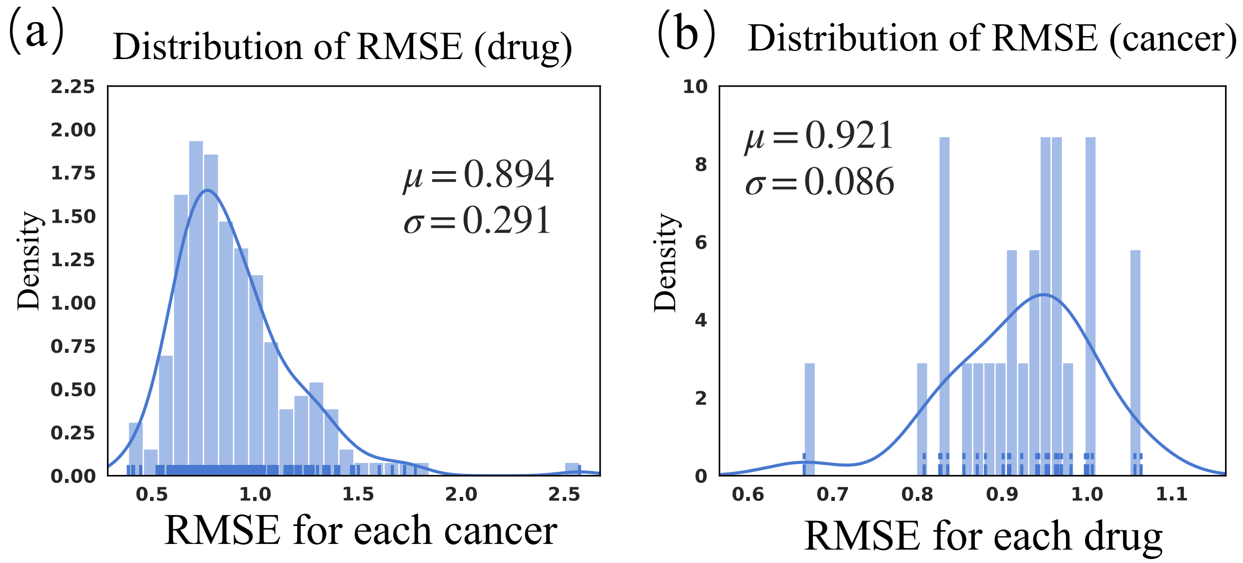

Supplementary Figure S7: **(a)** and **(b)** compares the change of PCC and RMSE when using the enlarged test sets from 10% to 70% of the data.

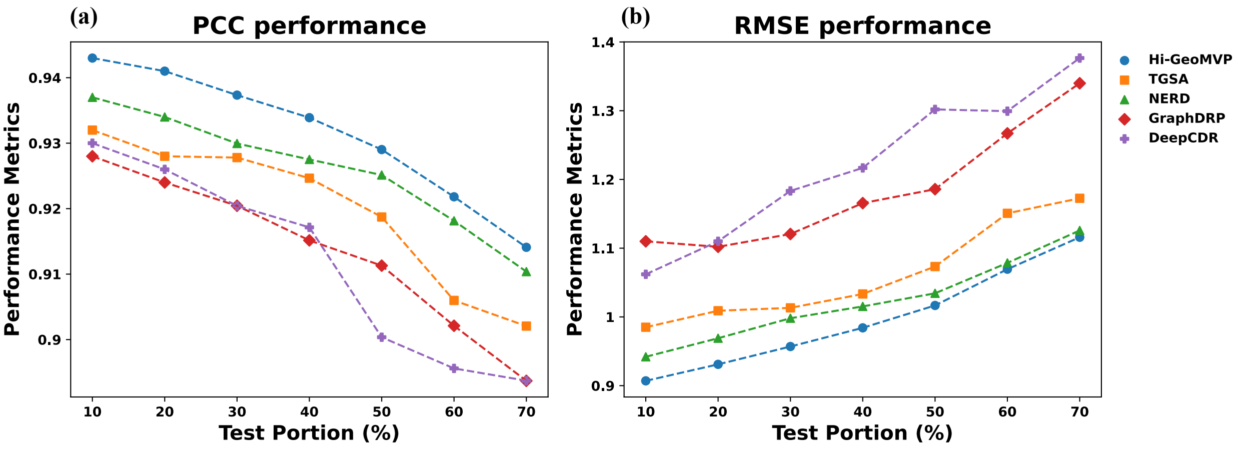

Supplementary Figure S8: The Cell-blind result in comparison for Hi-GeoMVP and TGSA for each cancer type. Each dot represents the mean PCC between predicted and actual log(IC50) values for a specific cancer type. The size of each dot is proportional to the number of samples for that particular cancer.

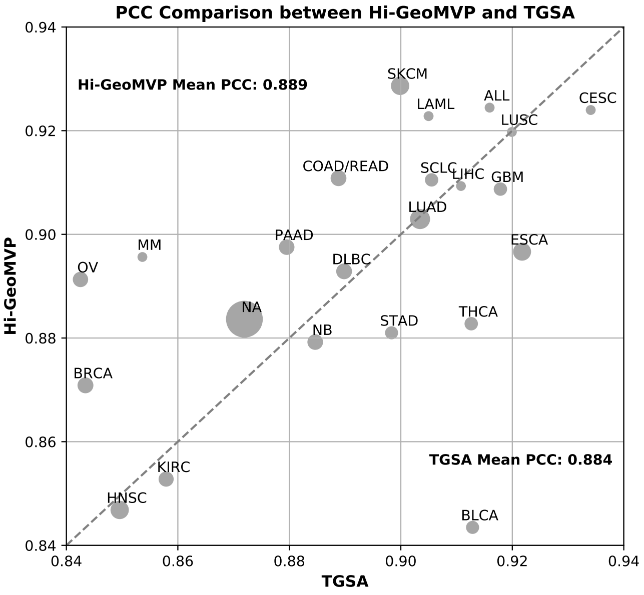

Supplementary Figure S9: Comparison of cell-blind results for Hi-GeoMVP and TGSA across different drugs. Each dot on the plot represents the mean PCC between the predicted and actual log(IC50) values for a particular drug. The overall p-value, calculated from the mean PCCs of all drugs, assesses the statistical significance of the observed differences.

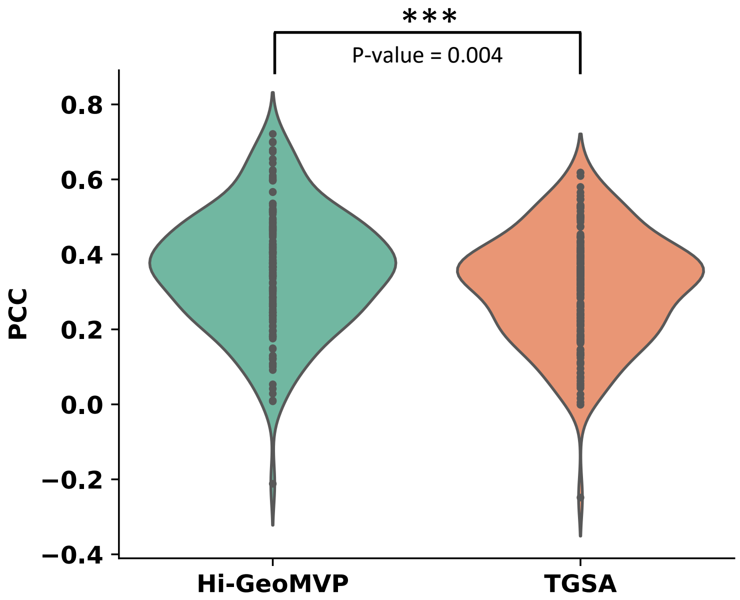

References

[1] Keyulu Xu, Weihua Hu, Jure Leskovec, and Stefanie Jegelka. How powerful are graph neural networks? In *ICLR, New Orleans, LA, USA*, 2019.

[2] Petar Veličković et al. Graph attention networks. In *ICLR, Vancouver, BC, Canada*. http://OpenReview.net, 2018.

[3] Diederik P Kingma and Max Welling. Auto-encoding variational bayes. In *ICLR, Banff, AB, Canada*. http://OpenReview.net, 2014.

[4] Justin Gilmer et al. Neural message passing for quantum chemistry. In *ICML 2017, Sydney, NSW, Australia*, volume 70, pages 1263–1272. PMLR, 2017.

[5] Xiaomin Fang et al. Geometry-Enhanced molecular representation learning for property prediction. *Nat. Mach. Intell.*, 4(2):127–134, February 2022.

[6] Lin Wang et al. Improved anticancer drug response prediction in cell lines using matrix factorization with similarity regularization. *BMC Cancer*, 17(1):513, August 2017.

[7] Weihua Hu et al. Strategies for pre-training graph neural networks. In *ICLR, Addis Ababa, Ethiopia*. http://OpenReview.net, 2020.

[8] Bin Chen et al. Comparison of random forest and pipeline pilot naïve bayes in prospective QSAR predictions. *J. Chem. Inf. Model.*, 52(3):792–803, March 2012.

[9] Jinyu Chen and Louxin Zhang. A survey and systematic assessment of computational methods for drug response prediction. *Brief. Bioinform.*, 22(1):232–246, January 2021.

[10] Chan Yeong Kim et al. HumanNet v3: An improved database of human gene networks for disease research. *Nucleic Acids Res.*, 50(D1):D632–D639, January 2022.

[11] Wei Jin et al. Node similarity preserving graph convolutional networks. In *Proceedings of the 14th ACM International Conference on Web Search and Data Mining*, WSDM '21, pages 148–156, New York, NY, USA, March 2021. Association for Computing Machinery.

[12] L et al. rdkit/rdkit: 2020_03_1 (q1 2020) release, March 2020.
